# Network Modeling of Crohn’s Disease Incidence

**DOI:** 10.1371/journal.pone.0156138

**Published:** 2016-06-16

**Authors:** Jean-Marc Victor, Gaëlle Debret, Annick Lesne, Leigh Pascoe, Pascal Carrivain, Gilles Wainrib, Jean-Pierre Hugot

**Affiliations:** 1 Laboratoire de Physique Théorique de la Matière Condensée, UMR 7600 Centre National de la Recherche Scientifique & Université Pierre et Marie Curie-Paris 6, Sorbonne Universités, Paris, France; 2 Institut de Génétique Moléculaire de Montpellier, Centre National de la Recherche Scientifique UMR 5535, Université de Montpellier, Montpellier, France; 3 Fondation Jean Dausset Centre d’Etude du Polymorphisme Humain, Paris, France; 4 Ecole Normale Supérieure, Paris, France; 5 Labex inflamex, Université Paris-Diderot Sorbonne Paris-Cité, Paris, France; 6 UMR 1149, Institut National de la Santé et de la Recherche Médicale, Paris, France; 7 Assistance Publique-Hôpitaux de Paris, Hôpital Robert Debré, Paris, France; BSRC 'Alexander FLEMING', GREECE

## Abstract

**Background:**

Numerous genetic and environmental risk factors play a role in human complex genetic disorders (CGD). However, their complex interplay remains to be modelled and explained in terms of disease mechanisms.

**Methods and findings:**

Crohn's Disease (CD) was modeled as a modular network of patho-physiological functions, each summarizing multiple gene-gene and gene-environment interactions. The disease resulted from one or few specific combinations of module functional states. Network aging dynamics was able to reproduce age-specific CD incidence curves as well as their variations over the past century in Western countries. Within the model, we translated the odds ratios (OR) associated to at-risk alleles in terms of disease propensities of the functional modules. Finally, the model was successfully applied to other CGD including ulcerative colitis, ankylosing spondylitis, multiple sclerosis and schizophrenia.

**Conclusion:**

Modeling disease incidence may help to understand disease causative chains, to delineate the potential of personalized medicine, and to monitor epidemiological changes in CGD.

## Introduction

Crohn's disease (CD) is a complex genetic disorder presumed to result from the interplay between susceptible genotypes and (still unknown) environmental risk factors in a given individual. Patients typically suffer from chronic diarrhea, abdominal pain and weight loss. CD seems to reflect a loss of immune tolerance of the host toward bacteria present in its digestive tract [1]. Several cell types present in the intestinal mucosa contribute to CD pathophysiology including epithelial cells, dendritic cells, lymphocytes, etc. As a whole, CD is characterized by an intestinal barrier dysfunction, an inflammation of the mucosa containing Th1/Th17 orientated T-cells and the development of fibrosis.

To date, genome-wide association studies (GWAS) have identified more than 140 CD susceptibility loci, which allowed the identification of biological pathways centrally involved in the disease [2]. The associated polymorphisms do not usually alter the peptidic chain of encoded proteins [3,4] but rather affect regulatory DNA sequences [5,6]. Most of the disease-associated polymorphisms exhibit odds ratios (OR) lower than 1.5 (ref 2). Search for common copy number variations through the genome reported limited significant associations [7]. Epistasis was also limited [8]. Finally, mutations with a strong phenotypic effect have been reported but in only a small number of CD patients with very early onset [9–11]. Thus, at the opposite of classic Mendelian disorders, all these findings support a diffuse causality in CD [12].

Diffuse causality is a well-known property of networks, here the biological network formed by CD susceptibility gene products [1,2,13]. It is now acknowledged that in many cases a network model is suitable to describe living systems. In such biological network models, physiological functions or molecular pathways are associated with network modules assumed to act independently [14,15]. The (patho-) physiological status of an organism may then be defined by the activity status of all the functional modules at a given time. We accordingly developed a network-based model of susceptibility to CD and derive from it an expression for the disease age-specific incidence rates and disease propensity of at-risk alleles.

## Methods

### A disease network model with functional modules

**Fig 1:**
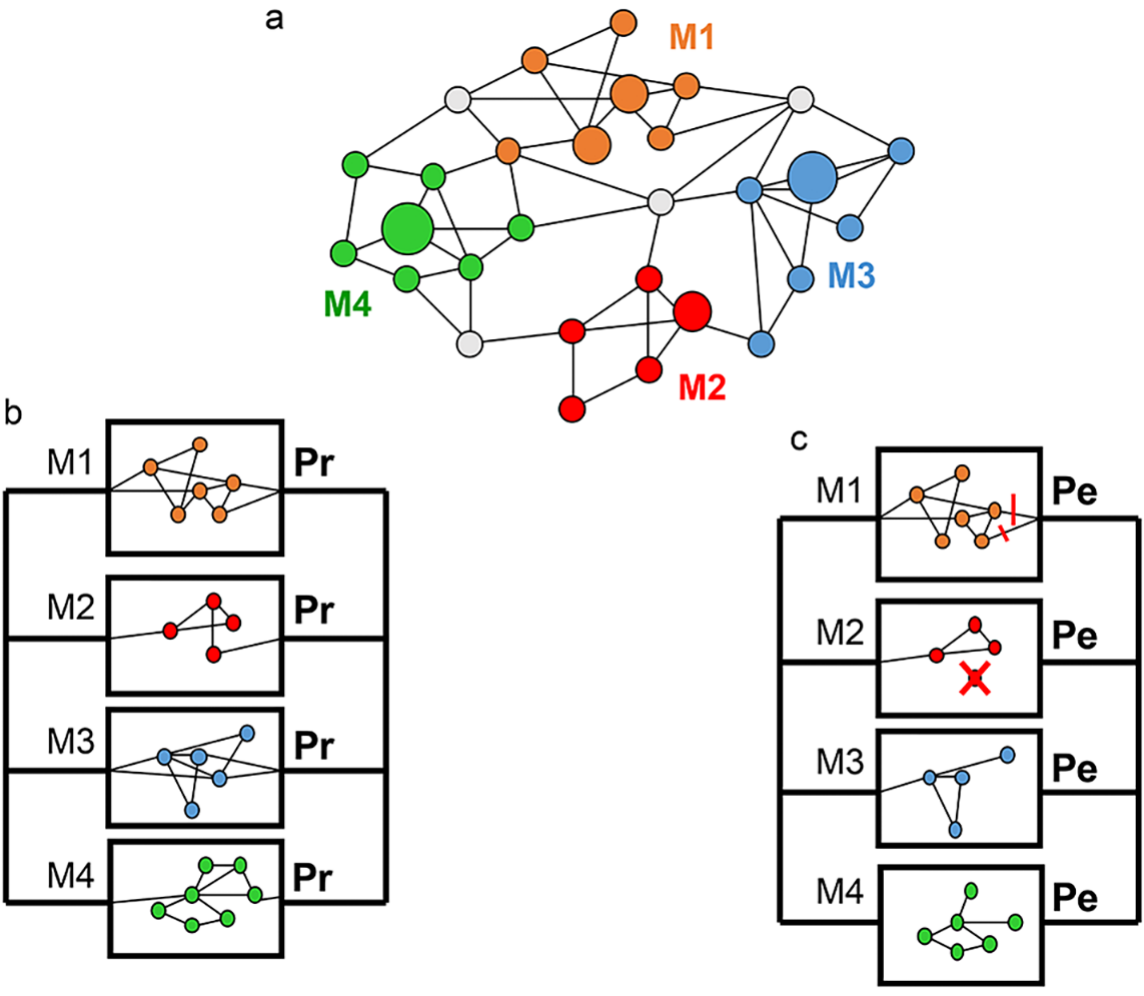
Schematic representation of the proposed model. (a). General representation of the mature biological network model. Circles represent functional elements playing a role in the biological network. These elements may be proteins, DNA regulatory sequences, small RNAs, metabolites, etc. Links denote physical or biochemical interactions and the circle size is proportional to the connectivity of the corresponding functional element. Nodes contributing to specific functional modules are represented by different colors (here the disease network is composed of four modules: M1 to M4). As an example, Nod2 is a node of the innate immune response module. Grey elements connect the different modules. Elements of the global network that are not involved in CD-associated functions are not represented. (b-c) Due to genetic, environmental and stochastic events, each module is in a protective (Pr) or permissive (Pe) state. (b) Most of the possible combinations of the functional states of the N modules are associated with health (here a single healthy combination is depicted) while (c) only one (or few) results in the CD phenotype. The protective/permissive states of each module are the result of many factors. Long-term environmental exposure may alter some modules (e.g cigarette smoking which may affect the intestinal permeability, module 1). Genetic mutations may also be deleterious for a given module (e.g. ATG16L1 mutations and autophagy, module 2). External factors may divert a functional element to alternative modules (e.g. the Yersinia effector YopJ affects NOD2 induced NF-kB activation in favor of interleukin-1b secretion, module 3). Finally stochastic events may also affect the structure and function of the modules with functional consequences (module 4).

For a given disease, we assume that only a limited number (N) of functional modules are pertinent to the disease status. In the case of CD plausible candidates are, for example, Th1/Th17 orientation of lymphocytes, intracellular autophagy or bacterial sensing [1]. This set of modules forms a sub-network (referred here to as the “CD network”) of the whole human biological network. The state of each module is either permissive for CD or protective against CD. The disease is assumed to occur when all of the modules involved in its pathology are in a permissive state (Fig 1).

The structure and activity of each module depend on environmental stimuli to which the organism is exposed. They are also influenced by constitutive structural and regulatory variations within the genes. Stochastic events may also contribute to the ontogenesis and activity of each module. As a whole, a functional state of a module must be seen as the first level of integration of the gene-environment interactions. A consequence of this model is that disease-associated risk alleles (respectively environmental risk factors) contribute more or less to the propensity of a particular module to become morbid, but they usually do not determine it entirely. The module structure and activity may thus vary from one individual to another, even among monozygotic twins. This feature agrees with the relatively low disease concordance rate among monozygotic twins in CD [16].

A delayed occurrence of the disease is the rule for many complex genetic disorders. As an example, CD usually occurs in young adults and appears exceptionally in the first years of life, indicating that -at least some- modules do not function in a disease-permissive state at birth. Many hypotheses may be invoked to explain this finding, including cumulative effects impacting the module function with time (e.g. immune response to enteric infections), exposure to environmental factors in adulthood only (e.g. cigarette smoking or alcohol consumption), the absence of specific modules in childhood (e.g. underdeveloped Peyer patches in the gut or absence of sexual hormones before puberty), etc. Whatever the causes, we thus assumed an ontogenetic period for the functional modules.

For simplification, we shall postulate below that each module is initially in a protective state but a more general model is presented in S1 File. Stabilization of the modules in a mature state occurs along the development of the organism. Whether the stabilized state is permissive or protective depends on both environmental exposures and structural or regulatory genetic variations (Fig 1). As CD is normally a life-long disease, the mature modules are assumed to stay in the adopted state for long periods. However, environmental and stochastic events may ultimately affect the functionality of the modules, with the possibility of subsequent conversion at a low rate. To model the evolution of the network activity after the ontogenesis of the modules, we adapted the model of organism longevity proposed by Gavrilov and Gavrilova and inspired from a general theory of system failure [17]. According to this model, death is a consequence of the aging of a network built with non-aging elements. By analogy, disease is viewed here as a consequence of the stochastic switch of a module from a protective state toward a disease-permissive state (note that the inverse change could also be seen with a consequent disease cure but these very rare cases are neglected in the following developments).

**Fig 2:**
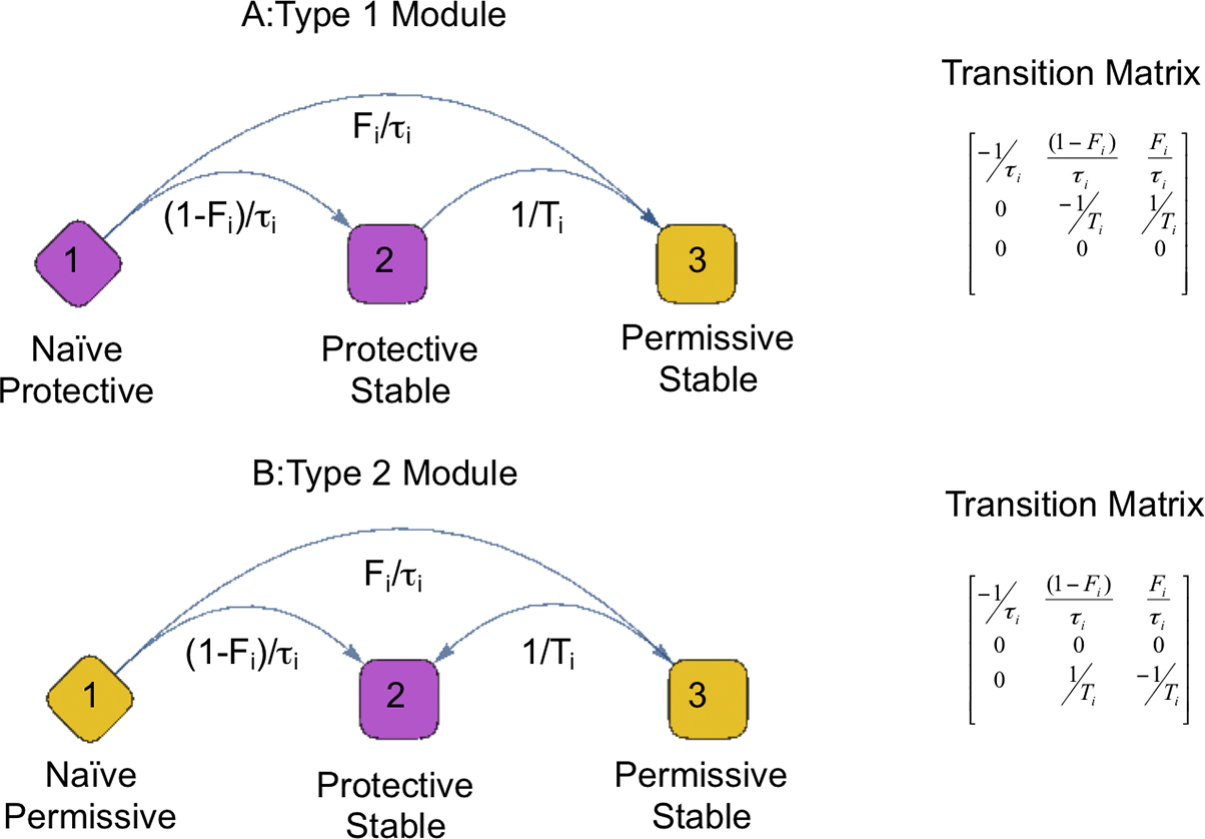
Module activity presented as a Continuous Markov Process. At birth each module is in a naive or immature state. Over time the modules stabilize into a state that can be protective or permissive for CD. The change of state is assumed to be an exponential process with rate parameter 1/τ_i_ and it may be towards a permissive or a protective state with probabilities F_i_ and (1−F_i_) respectively. Two types of modules are considered (S1 File). In the upper panel the module is protective in its naive state. We also allow for failure of the protective state 2 to the permissive state 3 as an exponential process with rate parameter 1/T_i_, assumed to be far slower than the initial process. In the lower panel we show modules that are permissive in their naive state. We allow failure from the permissive state to the protective state with rate parameter 1/T_i_. The corresponding transition matrices for the Markov Processes are shown on the right of the figure Diamond: unstable or naive state, Square: stable or mature state, Yellow color: permissive state, Purple color: protective state.

The state of a module at a given age can thus be modelled by a continuous Markov process with 3 states, as diagrammed in Fig 2. Transition of a module to its mature state is assumed to follow an exponential process with parameter *1/τ*_*i*_, while subsequent state conversion occurs at a constant rate *1/T*_*i*_ for the i^th^ module, with *T*_*i*_ far larger than *τ*_*i*_. The mature state may be permissive or protective, with probabilities *F*_*i*_ and *1-F*_*i*_ respectively. The probability *F*_*i*_ is referred to as the module disease propensity (MDP). On these bases, we derived the reliability *R*_*i*_*(x)* that the module *M*_*i*_ is still in a protective state at age *x* and the probability that CD arises at a given age (S1 File).

### Calculation of age-specific incidence rates

Under the assumption that each module is initially in a protective state, the probability that CD arises before age *x* can be written:

$$\mathrm{Pr}(CD|x)=\prod_{i=1}^{N}\left\{\left(1-\frac{1-F_{i}}{1-\frac{\tau_{i}}{T_{i}}}\right)\left(1-e^{-\frac{x}{\tau_{i}}}\right)+\frac{1-F_{i}}{1-\frac{\tau_{i}}{T_{i}}}\left(1-e^{-\frac{x}{T_{i}}}\right)\right\}$$ [1]

This general model has *3N+1* parameters, where *N* is the number of modules. To reduce the number of parameters and avoid over-fitting of the data, we made a so-called “homogenization" or “mean-field approximation”, whereby the values *τ*_*i*,_ *T*_*i*_ and *F*_*i*_ are replaced in each module (i.e. for each *i*) by their respective geometric means τ_,_ T and Φ. The probability that CD occurs before age *x* for this 4-parameter model is then written:

$$\mathrm{Pr}(CD|x)={\left\{\left(1-\frac{1-\Phi }{1-\frac{\tau }{T}}\right)\left(1-e^{-\frac{x}{\tau }}\right)+\frac{1-\Phi }{1-\frac{\tau }{T}}\left(1-e^{-\frac{x}{T}}\right)\right\}}^{N}$$ [2]

The age-specific incidence rate of CD is, by definition, equal to

$$I(x)=\frac{d}{dx}\mathrm{Pr}(CD|x)$$ [3]

which can be written as:

$$I(x)=N\left(\frac{\left(1-\frac{1-\Phi }{1-\frac{\tau }{T}}\right)e^{-\frac{x}{\tau }}}{\tau }+\frac{\left(1-\Phi \right)e^{-\frac{x}{T}}}{T\left(1-\frac{\tau }{T}\right)}\right){\left\{\left(1-\frac{1-\Phi }{1-\frac{\tau }{T}}\right)\left(1-e^{-\frac{x}{\tau }}\right)+\frac{1-\Phi }{1-\frac{\tau }{T}}\left(1-e^{-\frac{x}{T}}\right)\right\}}^{N-1}$$ [4]

This equation predicts an exponential increase followed by a peak and a slow decrease at advanced ages, as generally observed for age-specific CD incidence curves.

### Impact of genetic polymorphisms

We also investigated how OR of the disease-associated alleles, measured in GWAS studies, are related to our model. In the above analysis, MDPs were defined as averages over the whole population, notwithstanding genetic polymorphisms. To go beyond this simple analysis, we considered a polymorphism α with two alleles, one protective, α_P_, and the other, α_R_, at-risk for CD. The frequency of the at-risk allele α_R_ in the population (risk allele frequency, RAF) is denoted *p*_*α*_. For each module *M*_*i*_, we introduced the MDP *F*_*i*_*(α*_*R*_*)* over the subpopulation carrying the allele α_R_, (i.e. either the homozygote (α_R,_ α_R_) or heterozygote (α_R,_ α_P_) genotypes). In addition we denoted *F*_*i*_*(α*_*P*_*)* the MDP over the rest of the population, which genotype was (α_P,_ α_P_). We assumed that a given genetic locus α predominantly affects a single module *M*_*i(α)*_ among the *N* modules of the CD network, and thus simply denoted *F(α*_*R*_*)* the MDP of this module. For rare diseases like CD (for which the OR can be approximated by the relative risk), the OR of the at-risk variant at locus α can be expressed (S2 File) as a function of *p*_*α*_ and *F(α*_*R*_*)*:

$$OR(\alpha )\approx \frac{{\left(1-p_{\alpha }\right)}^{2}F\left(\alpha_{R}\right)}{\Phi -\left[1-{\left(1-p_{\alpha }\right)}^{2}\right]F\left(\alpha_{R}\right)}$$ [5]

## Results

### Fitting the age-dependent incidence curves for CD

**Fig 3:**
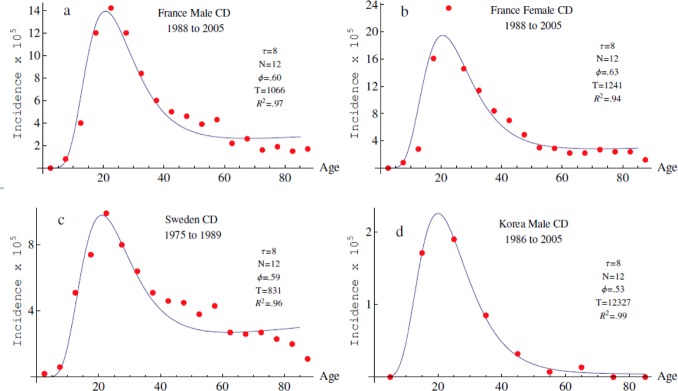
Age-specific CD incidence rates observed in several populations, compared with model predictions. Parameters *τ*, *T*, *N* and Φ were first estimated from several published age-specific incidence curves; the values of *τ* = 8 years and *N* = 12 were retained for the model and the estimated values of T and Φ updated. Fitted data sets in a) French male population-based register [18], b) Females from Northern France [18], c) Sweden [19] and d) Korean males [20]. Reported data are shown as red dots while the fitted theoretical curves are in blue.

Extensive fitting of the 4-parameter non-linear model to published data, using a quasi-Newton method to minimize squared residuals, gave excellent fits to the data, with the model explaining about 98% of the variance. Several sets of parameters well fitted the tested data sets with values of *τ*, *N*, Φ and *T* ranging respectively from 7y to 12y; 6 to 24; .38 to .79 and 1150y to 26300y. In all the tested data sets a model with 12 functional modules, with an expected mean time to stabilization *τ* of 8 years, gave among the best fits (Fig 3). This consistency was observed among sexes in a population-based registry from Northern France [18]. It was also observed in countries exhibiting very different disease prevalence rates (may be except for the oldest people in one dataset). In Sweden [19], where the disease is ancient and frequent, the values of Φ and T were slightly lower than those observed in France (Φ = 0.59 and T = 830 years) while in Korea [20], where the disease is rare and recent, the value of Φ was lower (0.53) with a ten-fold higher estimated value of T (12.300 years). Since the number of biological modules and their time of maturation are likely to be constant between populations, constancy between data sets was reassuring and we fixed these parameters (*N =* 12, *τ =* 8) in subsequent analyses. Of note, the best values of Φ were higher than 0.5 indicating that, on average, a module more often adopts a disease-permissive state once stabilized. Large values of T confirmed that the functional state of a module is a persistent life-long status in most people.

Of course, the model *per se* does not provide any information on the function of the module. However, in the report of the largest GWAS for CD, between 10 and 14 functional modules have been derived from genetic analyses^2^: inflammatory response, defence response to bacteria, IgG binding, innate immune response, T cell co-stimulation, B cell receptor signalling, cytokine-mediated signalling, interferon gamma-mediated signaling, T cell receptor complex, T cell activation and autophagy, ubiquitination and NF-kB, TGFβ signaling, and RORγt. It thus appears that the number of 12 modules proposed here is in good concordance with the literature.

### Consequences of environmental changes

**Fig 4:**
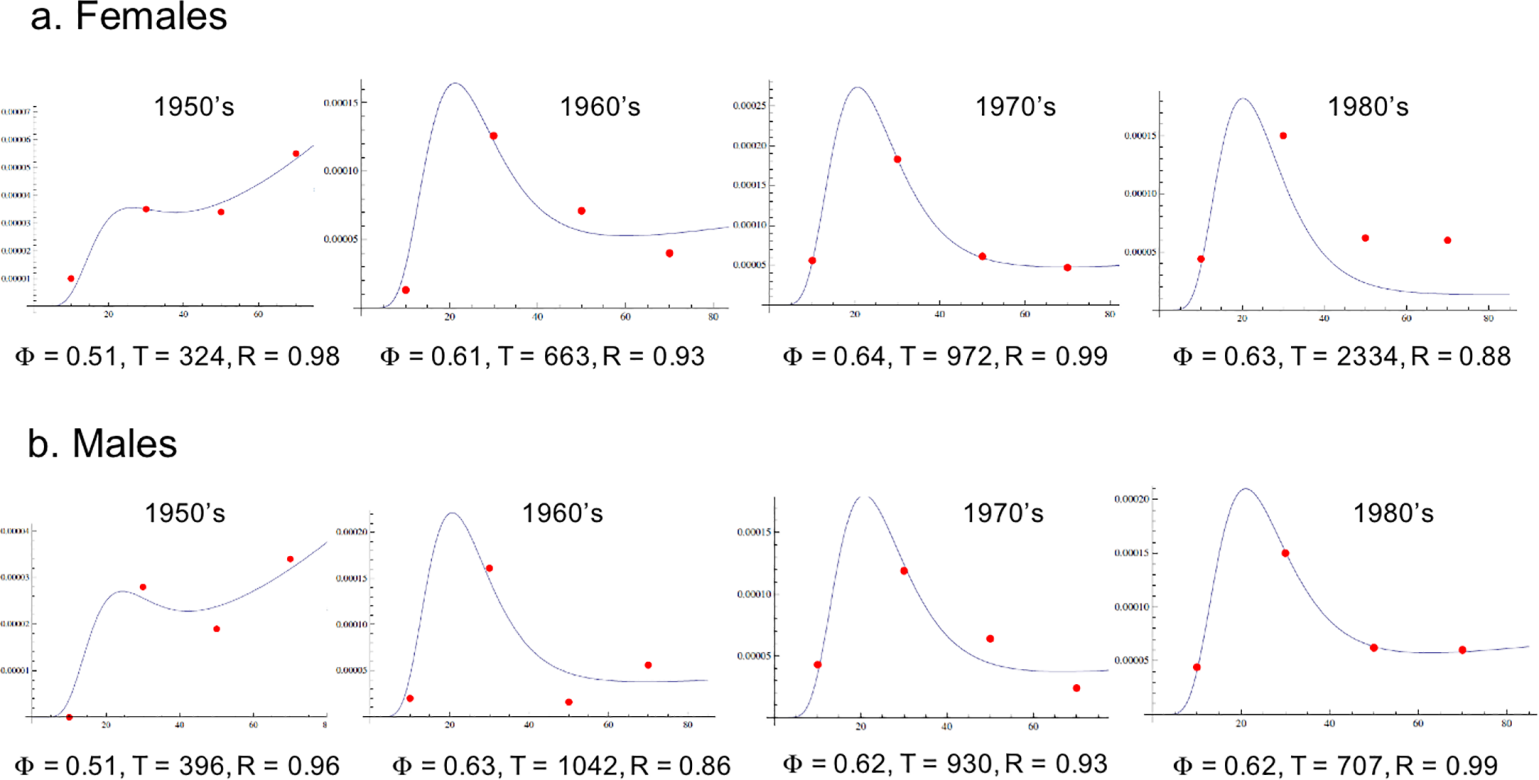
Evolution of the model parameters during the CD outbreak during the 20-th century in the USA. The annual standardized incidence rates were derived from ref. 12, which consists of a long-term epidemiological follow-up in Olmsted County, Minnesota, USA [22]. The measured incidence rates are shown in red for different decades while the modeled curves are indicated in blue (a) in females and (b) in males. The values of τ and N were fixed to τ = 8 years and N = 12 (see Fig 3). The optimized values for T and Φ are indicated for each dataset with the corresponding values of the correlation coefficient R between the dataset and the fitted model.

CD incidence increased significantly during the 20^th^ century in Western countries and many authors agree that this increase was caused by an environmental change associated with the modern occidental way of life [21]. Looking at data from Olmsted County Minnesota from 1950’s to 1980’s [22], we observed that the proposed model with *N* = 12 and *τ* = 8y remained valid in most cases, allowing to adjust *T* and Φ values for each decade (Fig 4). The obtained value of Φ increased from 0.51 to 0.63, consistently with the increased incidence of CD. *T* also increased from about 350y to 1000y suggesting that at the beginning of the outbreak, the occurrence of environmental risk factor(s) temporarily destabilized the functional modules toward the disease-permissive state with a subsequent transient decrease of *T*. Later, exposure of the whole population resulted in a re-stabilization of the modules with higher MDP values and again large *T* values.

**Fig 5:**
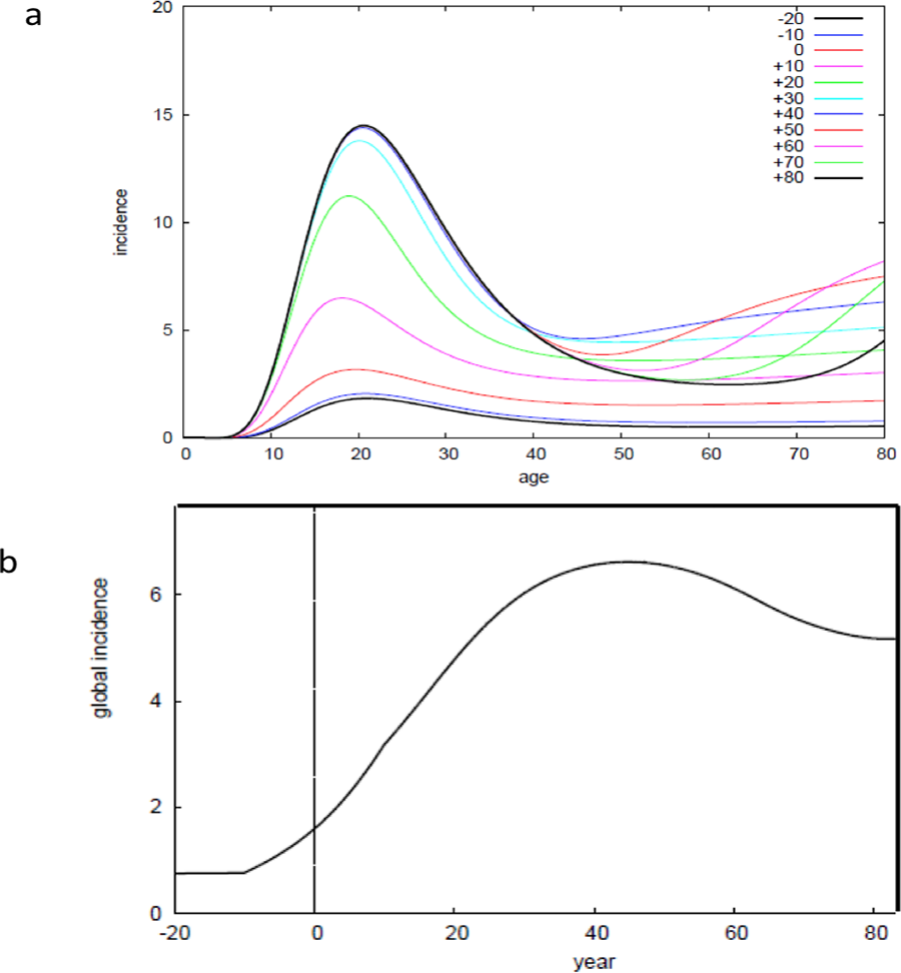
Evolution of the age-specific incidence curves following an environmental change. a) Using the transition model described in Fig A in S4 File, age-specific incidence curves were computed for several decades before and after the transition time *t*_*50*_ defined as the time of exposure of half of the population to a spreading environmental risk factor (it corresponds to reference 0 on the curves). Parameter values *τ* = 8y, *N* = 12, Φ_1_ = 0.51, Φ_2_ = 0.63, *T* = 1240y and T^*^ = 350y were derived from other datasets (Fig 3) b) Temporal variation of the global incidence rates computed from -20y and +80y around *t*_*50*_.

To further explore these findings, we investigated the impact of new environmental risk factor(s) on the age-specific incidence curves in our model (S3 and S4 Files). We assumed that an increasing proportion of the population was exposed to the new environmental risk factor(s) and computed the evolution of the modeled age-specific incidence curves for the decades around the time *t*_*5*0_ representing the moment where half of the population has been exposed to the risk factor(s). We used the parameters derived from the preceding analyses (*τ* = 8y, *N* = 12, Φ_before_ = 0.51, Φ_after_ = 0.63). Assuming a stable environment before and after the transition, *T* was set identical before and after the environmental changes (equal to the present value of 1240y). The parameter reflecting the duration of the transition did not notably affect the curves for a wide range of values (not shown). Under these conditions, the curves displayed an increasing incidence peak between the ages of 20y and 30y, which stabilized about 30y after t_50_ (Fig 5A). However, the effect of the new environmental risk factor(s) was difficult to detect before t_50_. A second peak was observed forty years after *t*_*50*_ in the oldest people, and disappeared after a few decades. This unexpected evolution of the age-specific incidence curves is in fact also observed in real long-term follow-up datasets [23].

Based on the computed age-specific incidence curves, we derived the annual incidence rates in the population from -20y to +80y around *t*_*50*_ (Fig 5B). The delayed capacity to detect the impact of the environmental factor was confirmed: less than a quarter of the maximum annual incidence rate over time was observed at t_50_. The incidence increased until year 40 after t_50_ with a small decrease thereafter. This secular trend was concordant with CD literature with often a global incidence increase during 3 or 4 decades followed by a small decrease [24].

Comparing the computed curves and the reported data on CD incidence during the 20^th^ century in Western countries, it was possible using our model to propose some dates corresponding to *t*_*50*_ and then to speculate on putative risk factors. CD was reported in 1932 by Crohn and colleagues in New-York [25]. It initially developed in white, urban, middle-class people and then extended to the whole population. Population-based data with long-term follow-up suggest that a quarter of the maximum incidence was reached in the 40’s in USA [22], in the 50’s in Sweden [19], in the 60’s in United Kingdom [26] and later in Southern Europe. At the same time, half of the population was equipped with a home refrigerator in these countries [27]. These observations further argue for the hypothesis of a role of refrigerated food in CD [28].

### Impact of at-risk alleles

**Fig 6:**
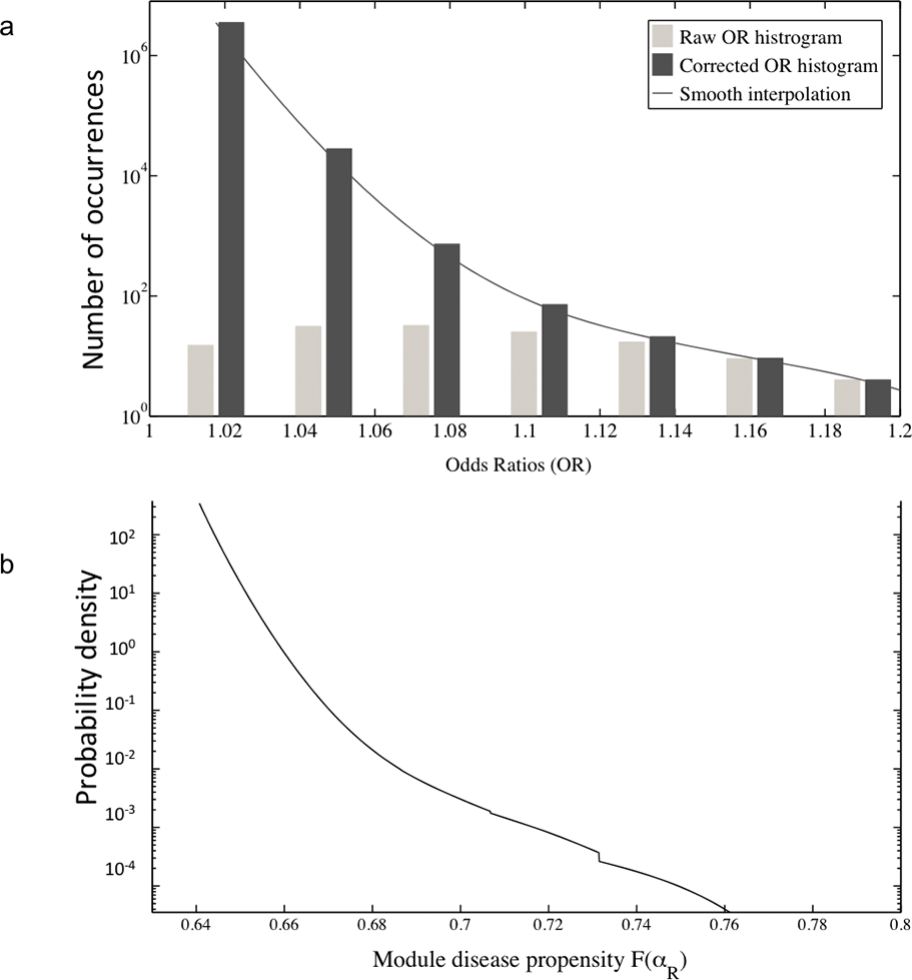
Impact of genetic polymorphisms on Module Disease Propensities (MDP). a) Histograms (in log-scale) of raw and corrected Odds Ratios corresponding to the recently published 140 CD-associated susceptibility loci [2]. b) Inferred probability distribution (in log-scale) over these loci of the MDP *F(α*_*R*_*)* corresponding to the at-risk variant *α*_*R*_ (see text) when considering an averaged value Φ = 0.63.

We also considered the impact of genetic variations on the fate of the modules of the CD network. The OR distribution corresponding to the dataset of 140 CD-associated risk alleles derived from GWAS displays a maximum value for OR≈1.1 (ref 2). However, the lowest values of OR are unavoidably under-represented due to inherent limitations of GWAS statistical power. We thus corrected for this bias (S5 File) and obtained a plausible estimation of the exact distribution ν of significant ORs (Fig 6A). Then we established from Eq 5 an explicit relationship between this distribution ν, the RAF distribution ρ, and the distribution *g* of the variables *F(α*_*R*_*)* over all loci (S6 File):

$$g(x)\approx \int \nu \left(\frac{x{\left(1-p\right)}^{2}}{\Phi -x(2p-p^{2})}\right)\frac{\Phi {\left(1-p\right)}^{2}}{{\left[\Phi -x(2p-p^{2})\right]}^{2}}\rho (p)dp$$ [6]

According to this formula the propensities *F(α*_*R*_*)* over all loci were very narrowly distributed in the vicinity of Φ (Fig 6B). Hence, the huge majority of all at-risk alleles are associated with nearly the same MDP, close to Φ, with no value higher than 0.75 (Fig 6B). Thus, at-risk alleles have each limited effects at the population scale, a finding which is in accordance with previous reports, even for the most at-risk alleles [29]. Of note, *F(α*_*R*_*)* is a population average of the distribution of individual propensities. As a comparison, for an allele causing a Mendelian trait (i.e. a disease with a single module network), *F(α*_*R*_*)* would be its penetrance. Interestingly, if for the huge majority of individuals, at-risk alleles have limited functional effects, this does not preclude the possibility that a very small fraction of allele carriers exhibits high individual MDPs corresponding to strong functional effects of the at-risk alleles.

### Application to other complex genetic disorders

**Fig 7:**
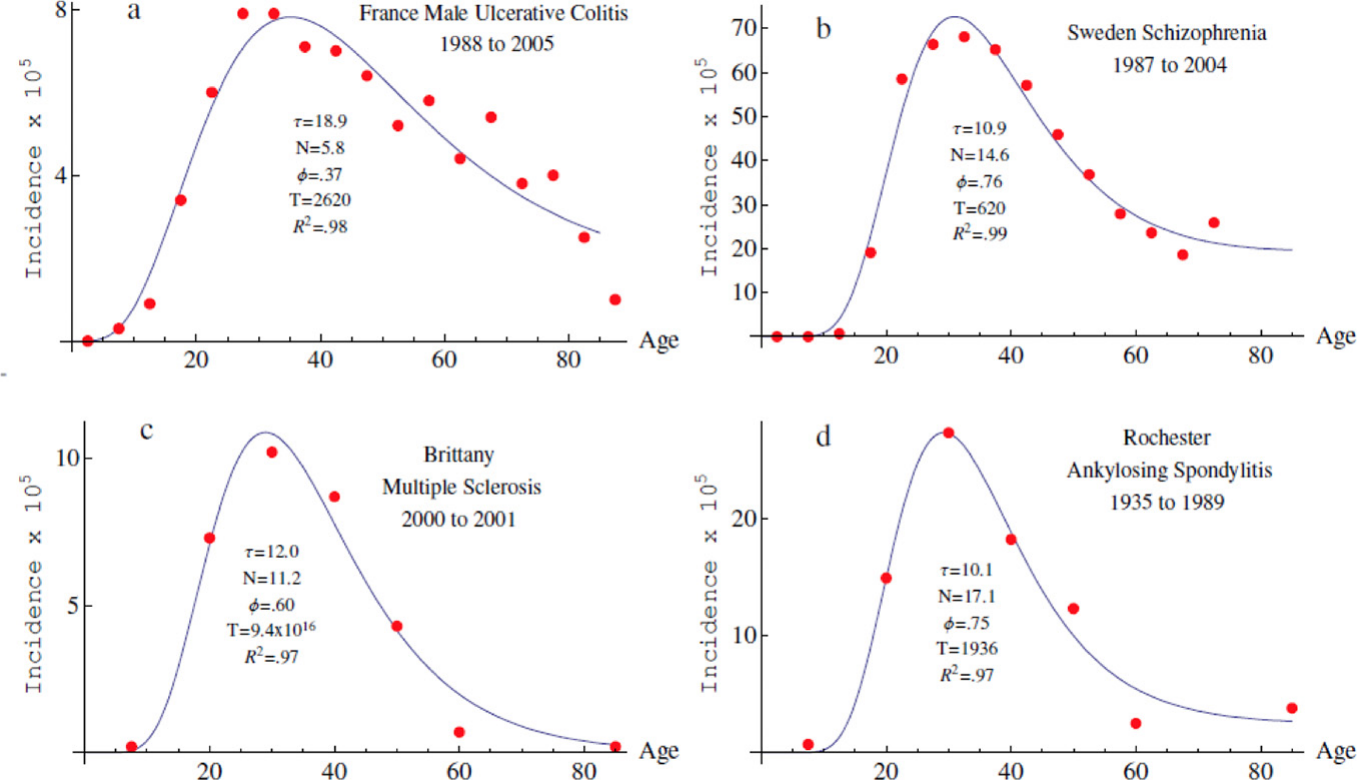
Application to other complex genetic disorders. The 4-parameter model was fitted to published datasets for (a) French male ulcerative colitis [18]; (b) schizophrenia [30]; (c) multiple sclerosis [31]; (d) ankylosing spondylitis [32]. Published data are shown as red dots and the computed curves as blue lines.

Complex genetic diseases are all characterized by an interaction between multiple genetic and environmental risk factors. For disorders mainly affecting the young adults, age-specific incidence curves most often resemble each other with an exponential increase toward a peak of incidence followed by a slower decrease of incidence in the oldest people (Fig 7). These diseases thus appear as good candidates for applying our model. (Note that, in contrast, for ageing-related and degenerative disorders, the curves are most often monotonously ascending and do not reach a peak. If this finding does not argue for the use of our model it does not discard the rationale underlying the proposed model for these disorders. It may only indicate that the age-incidence curves are truncated before the peak of incidence due to life expectancy of human beings in case of ageing-related and degenerative diseases).

We fitted the age-specific incidence curves available for ulcerative colitis (UC) [18], schizophrenia [30], multiple sclerosis [31] and ankylosing spondylarthritis [32]. The values of *τ* fluctuated from 10 to 19 years while the values of *N* fluctuated from 6 to 17. Interestingly, for UC, a lower number of modules than for CD was predicted. This could be seen contradictory with the fact that CD and UC share most of their susceptibility alleles. However, despite common genetic risk alleles, several functional modules like autophagy or innate immunity seem to be specific to CD and may thus explain the discrepancy. Finally, and as expected, for all the tested chronic diseases, *T* was always large. Overall, these results suggest that the proposed model also applies to other complex genetic conditions.

## Discussion

The model proposed here is based on the representation of biological functions as a modular network. The functional states of the modules are seen as random variables affected by gene-environment interactions. The disease is then defined by a limited number of modules, each in a given at-risk functional state. Aging dynamics of the functional network allows explaining epidemiological findings like the age-dependent incidence curves (and their variations across time and space) or the disease risk attributable to susceptibility alleles for CD and other complex genetic disorders.

The concept of biological network is now widely acknowledged by biologists. The modular nature of the biological networks is also widely accepted [15]. The main originality of our model is to integrate gene-environment interaction at the level of biological modules instead of at the level of the whole organism/network. In other words, the reaction norm defining the phenotype from its genetic background and its environmental exposure is displaced to a lower scale, which can be seen as a sub-phenotype. This way of thinking is logical if one reasons in terms of biological function, which is a direct consequence of functional states of cells or even molecules. The whole phenotype of an organism (here a morbid condition) thus needs to be dissected and analysed at lower levels and must be seen as a systemic property of a hierarchical network.

The proposed model strongly challenges the current reductionist understanding of disease causality. The phenotype is fully determined by the functional status of biological modules but the functional status of the modules themselves are not fully predictable. The only predictable thing is their respective MDPs, which are themselves a consequence of genetic, environmental and gene-environment parameters. However, MDPs are only propensities and it is thus impossible to fully predict the status of a given module, and by consequence of the module network. Accordingly, the disease is fully determined neither by the DNA sequence (the genome) nor by the exposure to environmental factors (the exposome) nor by any combination of genetic and environmental factors. Additional factors must be taken into account, namely stochastic events that draw CD-permissive or CD-protective modules randomly with their respective propensities. As a result, the model leads to an individual-centred notion of health, disease risk and preventive actions. This opinion is fully supported by the incomplete concordance rates between monozygotic twins (who share their genetic and environmental backgrounds) in most of complex genetic disorders.

Finally and more practically, the proposed model may be used as a tool for public health decision-makers. As shown for CD, overseeing the age-dependent incidence curves may help to follow the impact of environmental changes and to test the plausibility of putative risk factors on disease outbreaks.

## Supporting Information

S1 File (DOCX)

S2 File (DOCX)

S3 File (DOCX)

S4 File (DOCX)

S5 File (DOCX)

S6 File (DOCX)

## Abbreviations

CD: Crohn's disease
GWAS: Genome-wide association studies
MDP: Module disease propensity
OR: odds ratio
RR: relative risk

## References

[1] Khor B, Gardet A, Xavier RJ. Genetics and pathogenesis of inflammatory bowel disease. Nature. 2011;474: 307–317. 10.1038/nature10209 21677747 PMC3204665

[2] Jostins L, Ripke S, Weersma RK, Duerr RH, McGovern DP, Hui KY, et al Host-microbe interactions have shaped the genetic architecture of inflammatory bowel disease. Nature. 2012;491: 119–124. 10.1038/nature11582 23128233 PMC3491803

[3] Momozawa Y, Mni M, Nakamura K, Coppieters W, Almer S, Amininejad L, et al Resequencing of positional candidates identifies low frequency IL23R coding variants protecting against inflammatory bowel disease. Nat Genet. 2011;43: 43–47. 10.1038/ng.733 21151126

[4] Beaudoin M, Goyette P, Boucher G, Lo KS, Rivas MA, Stevens C, et al Deep resequencing of GWAS loci identifies rare variants in CARD9, IL23R and RNF186 that are associated with ulcerative colitis. PLoS Genet. 2013;9: e1003723 10.1371/journal.pgen.1003723 24068945 PMC3772057

[5] Brest P, Lapaquette P, Souidi M, Lebrigand K, Cesaro A, Vouret-Craviari V, et al A synonymous variant in IRGM alters a binding site for miR-196 and causes deregulation of IRGM-dependent xenophagy in Crohn's disease. Nat Genet. 2011;43: 242–245. 10.1038/ng.762 21278745

[6] Maurano MT, Humbert R, Rynes E, Thurman RE, Haugen E, Wang H, et al Systematic localization of common disease-associated variation in regulatory DNA. Science. 2012;337: 1190–1195. 10.1126/science.1222794 22955828 PMC3771521

[7] Wellcome Trust Case Control Consortium, Craddock N, Hurles ME, Cardin N, Pearson RD, Plagnol V, et al Genome-wide association study of CNVs in 16,000 cases of eight common diseases and 3,000 shared controls. Nature. 2010;464: 713–720. 10.1038/nature08979 20360734 PMC2892339

[8] Wei Z, Wang W, Bradfield J, Li J, Cardinale C, Frackelton E, et al Large sample size, wide variant spectrum, and advanced machine-learning technique boost risk prediction for inflammatory bowel disease. Am J Hum Genet. 2013;92: 1008–1012. 10.1016/j.ajhg.2013.05.002 23731541 PMC3675261

[9] Glocker EO, Kotlarz D, Boztug K, Gertz EM, Schäffer AA, Noyan F, et al Inflammatory bowel disease and mutations affecting the interleukin-10 receptor. N Engl J Med. 2009;361: 2033–2045. 10.1056/NEJMoa0907206 19890111 PMC2787406

[10] Muise AM, Xu W, Guo CH, Walters TD, Wolters VM, Fattouh R, et al NADPH oxidase complex and IBD candidate gene studies: identification of a rare variant in NCF2 that results in reduced binding to RAC2. Gut. 2012;61: 1028–1035. 10.1136/gutjnl-2011-300078 21900546 PMC3806486

[11] Aguilar C, Lenoir C, Lambert N, Bègue B, Brousse N, Canioni D, et al Characterization of Crohn disease in X-linked inhibitor of apoptosis-deficient male patients and female symptomatic carriers. J Allergy Clin Immunol. 2014;134: 1131–41. 10.1016/j.jaci.2014.04.031 24942515

[12] Debret G, Jung C, Hugot JP, Pascoe L, Victor JM, Lesne A. Genetic susceptibility to a complex disease: the key role of functional redundancy. Hist Philos Life Sci. 2011;33: 497–514. 22662507

[13] Rossin EJ, Lage K, Raychaudhuri S, Xavier RJ, Tatar D, Benita Y, et al Proteins encoded in genomic regions associated with immune-mediated disease physically interact and suggest underlying biology. PLoS Genetics. 2011;7: e1001273 10.1371/journal.pgen.1001273 21249183 PMC3020935

[14] Papin JA, Hunter T, Palsson BO, Subramaniam S. Reconstruction of cellular signalling networks and analysis of their properties. Nat Rev Mol Cell Biol. 2005;6: 99–111. 15654321 10.1038/nrm1570

[15] Barabasi AL, Gulbahce N, Loscalzo J. Network medicine: a network-based approach to human disease. Nature Reviews Genetics. 2011;12: 56–68. 10.1038/nrg2918 21164525 PMC3140052

[16] Gordon H, Trier Moller F, Andersen V, Harbord M. Heritability in inflammatory bowel disease: from the first twin study to genome-wide association studies. Inflamm Bowel Dis. 2015;21: 1428–1434. 10.1097/MIB.0000000000000393 25895112 PMC4450891

[17] Gavrilov LA, Gavrilova NS. The reliability theory of aging and longevity. J Theor Biol. 2001;213: 527–545. 11742523 10.1006/jtbi.2001.2430

[18] Chouraki V, Savoye G, Dauchet L, Vernier-Massouille G, Dupas JL, Merle V, et al The changing pattern of Crohn's disease incidence in northern France: a continuing increase in the 10- to 19-year-old age bracket (1988–2007). Aliment Pharmacol Ther. 2011;33: 1133–1142. 10.1111/j.1365-2036.2011.04628.x 21488915

[19] Lapidus A, Bernell O, Hellers G, Persson PG, Löfberg R. Incidence of Crohn's disease in Stockholm County 1955–1989. Gut. 1997;41: 480–486. 9391246 10.1136/gut.41.4.480 PMC1891542

[20] Yang SK, Yun S, Kim JH, Park JY, Kim HY, Chang DK, et al Epidemiology of inflammatory bowel disease in the Songpa-Kangdong district, Seoul, Korea, 1986–2005: a KASID study. Inflamm Bowel Dis. 2008;14: 542–549. 17941073 10.1002/ibd.20310

[21] Cosnes J, Gower-Rousseau C, Seksik P, Cortot A. Epidemiology and natural history of inflammatory bowel diseases. Gastroenterology. 2011;140: 1785–1794. 10.1053/j.gastro.2011.01.055 21530745

[22] Loftus CG, Loftus EV Jr, Harmsen WS, Zinsmeister AR, Tremaine WJ, Melton LJ 3rd, et al Update on the incidence and prevalence of Crohn's disease and ulcerative colitis in Olmsted County, Minnesota, 1940–2000. Inflamm Bowel Dis. 2007;13: 254–61. 17206702 10.1002/ibd.20029

[23] Rose JD, Roberts GM, Williams G, Mayberry JF, Rhodes J. Cardiff Crohn's disease jubilee: the incidence over 50 years. Gut. 1988;29: 346–51. 3356366 10.1136/gut.29.3.346 PMC1433600

[24] Molodecky NA, Soon IS, Rabi DM, Ghali WA, Ferris M, Chernoff G, et al Increasing incidence and prevalence of the inflammatory bowel diseases with time, based on systematic review. Gastroenterology. 2012;142: 46–54. 10.1053/j.gastro.2011.10.001 22001864

[25] Crohn BB, Ginzburg L, Oppenheimer GD. Landmark article Oct 15, 1932. Regional ileitis. A pathological and clinical entity. By Burril B. Crohn, Leon Ginzburg, and Gordon D. Oppenheimer. JAMA. 1984;251: 73–79. 6361290 10.1001/jama.251.1.73

[26] Gunesh S, Thomas GA, Williams GT, Roberts A, Hawthorne AB. The incidence of Crohn's disease in Cardiff over the last 75 years: an update for 1996–2005. Aliment Pharmacol Ther. 2008;27: 211–219. 18005244 10.1111/j.1365-2036.2007.03576.x

[27] Thévenot R. Essai pour une histoire du froid artificiel dans le monde. Institut International du froid, Paris (1978).

[28] Hugot JP, Alberti C, Berrebi D, Bingen E, Cézard JP. Crohn's disease: the cold chain hypothesis. Lancet. 2003;362: 2012–2015. 14683664 10.1016/S0140-6736(03)15024-6

[29] Hugot JP, Chamaillard M, Zouali H, Lesage S, Cézard JP, Belaiche J, et al Association of NOD2 leucine-rich repeat variants with susceptibility to Crohn's disease. Nature. 2001;411: 599–603. 11385576 10.1038/35079107

[30] Li X, Sundquist J, Sundquist K. Age-specific familial risks of psychotic disorders and schizophrenia: A nation-wide epidemiological study from Sweden", Schizophr Res. 2007,97: 43–50. 17933494 10.1016/j.schres.2007.09.027 PMC2225525

[31] Phadke JG, Downie AW. Epidemiology of multiple sclerosis in the north-east (Grampian region) of Scotland-an update. J Epidemiol Community Health. 1987;41: 5–13. 3668459 10.1136/jech.41.1.5 PMC1052567

[32] Carbone LD, Cooper C, Michet CJ, Atkinson EJ, O'Fallon WM, Melton LJ 3rd. Ankylosing spondylitis in Rochester, Minnesota, 1935–1989. Is the epidemiology changing? Arthritis Rheum. 1992;35: 1476–1482. 1472124 10.1002/art.1780351211

