# Supplementary material for: Network Modeling of Crohn’s Disease Incidence
Source: PLoS One. 2016 Jun 16;11(6):e0156138. doi: 10.1371/journal.pone.0156138 (PMC4911211; doi:10.1371/journal.pone.0156138)
Supplement: S1 File — (DOCX) [file pone.0156138.s001.docx]

**S1 File. Computation of the probability that the module $M_{i}$ is disease-permissive at age x and derivation of the probability that CD arises before age x.**

Modules are assumed to exist in a naïve state at birth and to adopt their mature function stochastically according to an exponential process (figures 1 and 2). Thus the probability of still being naïve at age x is $e^{-x/\tau_{i}}$ and of being mature is $(1-e^{-x/\tau_{i}})$. In any module we say that its state is permissive if it permits CD to occur and protective if it prevents the disease. CD is assumed to occur when all modules are in a permissive state.

There are two types of modules corresponding to those in which maturation of the module is necessary to be permissive and those that are permissive when naïve. In the first type of modules the naïve state is protective and maturation into a permissive or protective stable state occurs with probabilities F_i_ and (1-F_i_) respectively, where F_i_ is known as the module disease propensity (MDP). We moreover model failure of the stable protective state over the long term as an exponential process with parameter $1/T_{i}$. This leads to the continuous Markov process and associated transition matrix between the 3 functional states shown in figure 2A. From the continuous Markov model we can directly derive the 3 state probabilities for the i^th^ module as

$\Pr(1|x,i)=e^{-\frac{x}{\tau_{i}}}$ [S1]

$\Pr(2|x,i)=\frac{(1-F_{i})T_{i}e^{-\frac{x}{T_{i}}-\frac{x}{\tau_{i}}}\left(e^{\frac{x}{\tau_{i}}}-e^{\frac{x}{T_{i}}}\right)}{T_{i}-\tau_{i}}$ [S2]

$\Pr(3|x,i)=\frac{e^{-\frac{x}{T_{i}}-\frac{x}{\tau_{i}}}\left(T_{i}e^{\frac{x}{T_{i}}+\frac{x}{\tau_{i}}}-T_{i}e^{\frac{x}{\tau_{i}}}-F_{i}T_{i}e^{\frac{x}{T_{i}}}+F_{i}T_{i}e^{\frac{x}{\tau_{i}}}+\tau_{i}e^{\frac{x}{T_{i}}}-\tau_{i}e^{\frac{x}{T_{i}}+\frac{x}{\tau_{i}}}\right)}{T_{i}-\tau_{i}}=1-Pr\left(1|x,i\right)-Pr\left(2|x,i\right)$ [S3]

Now the probability for the i^th^ module to be in a permissive state is simply

$$\Pr(Permissive|x,i)=1-\Pr(1|x,i)-\Pr(2|x,i)$$

In the second type of modules, the module is permissive in the naïve state. Again the module can be matured into a permissive or protective state, but in this case long-term failure is from permissive to protective state. The state probabilities for the j^th^ type 2 module are written as:

$\Pr(1|x,j)=e^{-\frac{x}{\tau_{j}}}$ [S4]

$\Pr(2|x,j)=1-\Pr(1|x,j)-\Pr(3|x,j)$ [S5]

where

$\Pr(3|x,j)=\frac{e^{-\frac{x}{T_{j}}-\frac{x}{\tau_{j}}}\left(e^{\frac{x}{\tau_{j}}}-e^{\frac{x}{T_{j}}}\right)F_{j}T_{j}}{T_{j}-\tau_{j}}$ [S6]

and the probability of being in a permissive state is

$$\Pr(Permissive|x,j)=(1-\Pr(2|x,j))$$

Now we assume that CD occurs when all modules are in a permissive state. So if we have N_1_ type1 modules and N_2_ type 2 modules in the CD network, the probability of CD arising before age x can be written as:

$\Pr(CD|x)=\prod_{1=1}^{N1} \Pr(Permissive|x,i)\prod_{j=1}^{N2} \Pr(Permissive|x,j)$ [S7]

This expression can be simplified to

$\Pr(CD|x)=\prod_{i=1}^{N_{1}} \left\{\left(1-\frac{1-F_{i}}{1-\frac{\tau_{i}}{T_{i}}}\right)\left(1-e^{-\frac{x}{\tau_{i}}}\right)+\frac{1-F_{i}}{1-\frac{\tau_{i}}{T_{i}}}\left(1-e^{-\frac{x}{T_{i}}}\right)\right\}\prod_{j=N_{1}+1}^{N1+N2} \left\{\left(1-\frac{F_{j}}{1-\frac{\tau_{j}}{T_{j}}}\right)e^{-\frac{x}{\tau_{j}}}+\frac{F_{j}}{1-\frac{\tau_{j}}{T_{j}}}e^{-\frac{x}{T_{j}}}\right\}$ [S8]

which is the desired probability.
