# Supplementary material for: Network Modeling of Crohn’s Disease Incidence
Source: PLoS One. 2016 Jun 16;11(6):e0156138. doi: 10.1371/journal.pone.0156138 (PMC4911211; doi:10.1371/journal.pone.0156138)
Supplement: S2 File — (DOCX) [file pone.0156138.s002.docx]

**S2 File. Relation between the relative risks (RR) and the module disease propensities (MDP).**

Consider a single nucleotide polymorphism (SNP) with two alleles, one protective, α_P_, and the other, α_R_, at-risk for CD. The frequency of the at-risk allele α_R_ in the population (risk allele frequency, RAF) is denoted *p_α_*. There are three genotypes: (α_R_, α_R_), (α_R_, α_P_), (α_P_, α_P_) respectively homozygotes at risk, heterozygotes and homozygotes protective. Their respective probabilities of occurrence are $p_{\alpha}^{2}$, $2p_{\alpha}(1-p_{\alpha})$ and $(1-p_{\alpha})^{2}$.We moreover denote their respective MDPs as:

$\left\{\begin{matrix} F_{i}(\alpha_{R},\alpha_{R})=\Pr ob\left[M_{i}\text{ perm}\left|(\alpha_{R},\alpha_{R})\right.\right] \\ F_{i}(\alpha_{R},\alpha_{P})=\Pr ob\left[M_{i}\text{ perm}\left|(\alpha_{R},\alpha_{P})\right.\right] \\ F_{i}(\alpha_{P},\alpha_{P})=\Pr ob\left[M_{i}\text{ perm}\left|(\alpha_{P},\alpha_{P})\right.\right] \end{matrix}\right.$ [S24]

Remembering that F_i_ is the probability that the mature state of module M_i_ is permissive for CD and using the formula of total probabilities we obtain:

$p_{\alpha}^{2}F_{i}\left(\alpha_{R},\alpha_{R}\right)+2p_{\alpha}\left(1-p_{\alpha}\right)F_{i}\left(\alpha_{R},\alpha_{P}\right)+(1-p_{\alpha})^{2}F_{i}\left(\alpha_{P},\alpha_{P}\right)=F_{i}$ [S25]

We further partition the population into people with a genotype containing the at-risk α_R_ allele and people without this allele, i.e. homozygote (α_P_, α_P_) people. Hence we get two subsets, respectively denoted R and P, with respective occurrences $1-(1-p_{\alpha})^{2}$ and $(1-p_{\alpha})^{2}$. These subsets have respective MDPs equal to $F_{i}\left(\alpha_{R}\right)$ and $F_{i}\left(\alpha_{P}\right)$ that are related to $F_{i}(\alpha_{R},\alpha_{R})$, $F_{i}(\alpha_{R},\alpha_{P})$ and $F_{i}(\alpha_{P},\alpha_{P})$ through:

$$\left\{\begin{matrix} \left[1-(1-p_{\alpha})^{2}\right]F_{i}\left(\alpha_{R}\right)=p_{\alpha}^{2}F_{i}\left(\alpha_{R},\alpha_{R}\right)+2p_{\alpha}\left(1-p_{\alpha}\right)F_{i}\left(\alpha_{R},\alpha_{P}\right) \\ F_{i}\left(\alpha_{P}\right)=F_{i}\left(\alpha_{P},\alpha_{P}\right) \end{matrix}\right.$$

so that

$$\left[1-(1-p_{\alpha})^{2}\right]F_{i}\left(\alpha_{R}\right)+(1-p_{\alpha})^{2}F_{i}\left(\alpha_{P}\right)=F_{i}$$

Therefore

$F_{i}\left(\alpha_{P}\right)=\left[F_{i}-\left[1-(1-p_{\alpha})^{2}\right]F_{i}\left(\alpha_{R}\right)\right]/\left(1-p_{\alpha}\right)^{2}$ [S26]

Assuming that all *F_i_* are equal to Φ, we obtain:

$\left[1-(1-p_{\alpha})^{2}\right]F_{i}\left(\alpha_{R}\right)+(1-p_{\alpha})^{2}F_{i}\left(\alpha_{P}\right)=\Phi$ [S27]

(However note that $F_{i}\left(\alpha_{R}\right)$ are different for different *i*).

Therefore,

$F_{i}\left(\alpha_{P}\right)=\left[\Phi-\left[1-(1-p_{\alpha})^{2}\right]F_{i}\left(\alpha_{R}\right)\right]/\left(1-p_{\alpha}\right)^{2}$ [S28]

It is then possible to evaluate the Relative Risk (RR) of the at-risk variant at locus $\alpha$:

$RR(\alpha)=\prod_{i=1}^{N} \frac{F_{i}\left(\alpha_{R}\right)}{F_{i}\left(\alpha_{P}\right)}=\prod_{i=1}^{N} \frac{\left(1-p_{\alpha}\right)^{2}F_{i}\left(\alpha_{R}\right)}{\Phi-\left[1-\left(1-p_{\alpha}\right)^{2}\right]F_{i}\left(\alpha_{R}\right)}$ [S29]

Assuming that a single locus α predominantly affects a single module $M_{i_{\alpha}}$, the above product is dominated by the term corresponding to module $M_{i_{\alpha}}$, the $N-1$ other ratios being close to 1:

$RR(\alpha)\approx\frac{F_{i_{\alpha}}\left(\alpha_{R}\right)}{F_{i_{\alpha}}\left(\alpha_{P}\right)}$ [S30]

Using Eq. S27 and defining $F\left(\alpha_{R}\right)=F_{i_{\alpha}}\left(\alpha_{R}\right)$ we get:

$RR(\alpha)\approx\frac{\left(1-p_{\alpha}\right)^{2}F\left(\alpha_{R}\right)}{\Phi-\left[1-\left(1-p_{\alpha}\right)^{2}\right]F\left(\alpha_{R}\right)}$ [S31]

For rare diseases like CD, RR are close to OR, so that we finally get:

$OR(\alpha)\approx\frac{\left(1-p_{\alpha}\right)^{2}F\left(\alpha_{R}\right)}{\Phi-\left[1-\left(1-p_{\alpha}\right)^{2}\right]F\left(\alpha_{R}\right)}$ [S31 bis]
