# Supplementary material for: Network Modeling of Crohn’s Disease Incidence
Source: PLoS One. 2016 Jun 16;11(6):e0156138. doi: 10.1371/journal.pone.0156138 (PMC4911211; doi:10.1371/journal.pone.0156138)
Supplement: S3 File — (DOCX) [file pone.0156138.s003.docx]

**S3 File**. **Computation of the age-dependent incidence rate in a sharply varying environment.**

In order to compute the impact of an environmental change (from period E1 to period E2) on the age-specific incidence rate of the disease, we first considered a sharp transition occurring at a fixed time and how it affects the health of individuals of age *x* at time t (hence ). People were either born after the transition has occurred or they have passed through the transition period from $E_{1}$ to $E_{2}$.

To model the impact of the environmental change on both parameters Φ and T we assumed:

(i) the MDP $F_{i}$ of each module $M_{i}$ changes from $F_{i,1}$ to $F_{i,2}$ as the environment changes from $E_{1}$ to $E_{2}$ so that the geometrical mean Φ of the *N* modules changes from Φ_1_ to Φ_2_.

(ii) the value of the aging lifetime *transiently* changes from $T$ to $T^{*}$ as the environment changes from $E_{1}$ to $E_{2}$ *only* for those modules that are already stabilized in the original environment $E_{1}$. For the other modules, still immature, the aging lifetime is set to the original value $T$. The rationale behind assumption (ii) is that the network may adapt to the new environment so far as concerns the immature modules. When these immature modules get stabilized in the new environment $E_{2}$ they are as robust to ageing as the former modules were in the original environment $E_{1}$.

Let us call the probability that the module is protective at time *t* for people of age *x* (hence born at time *t-x*). In case of a module that is protective when naïve, is equal to the sum of the probability to be still in the naive state at age *x*, namely , and of the probability to be stabilized in the protective state at age *x* (hence at time *t*). In order to compute , we first derived an equation for , the probability that the module has been stabilized in the protective state before time *u* and is still in this state at time *u* (with ) for people born at time .

Note that with

(i) the probability that the module has been stabilized *before* time and is still in the protective state at time *u*,

(ii) the probability that the module has been stabilized *after* time and is still in the protective state at time *u*.

Both functions and are obtained in the following way:

1. if then and is solution of

$\frac{\partial f_{i}}{\partial u}=\frac{1-F_{i,1}}{\tau_{i}}e^{-\left[u-(t-x)\right]/\tau_{i}}-\frac{f_{i}}{T}$ [S9]

1. if then and are solutions of

$\frac{\partial f_{i}}{\partial u}=-\frac{f_{i}}{T^{*}}$ [S10]

$\frac{\partial g_{i}}{\partial u}=\frac{1-F_{i,2}}{\tau_{i}}e^{-\left[u-(t-x)\right]/\tau_{i}}-\frac{g_{i}}{T}$ [S11]

Remark: in Eq. S9 and Eq. S11 the terms depending on τ_i_ are the entering fluxes that contribute to increasing the probability that M_i_ is stabilized in the protective state .and the terms depending on *T* (*T*_1_ in Eq. S9 and Eq. S11, *T*_2_ in Eq. S10) are the exiting fluxes that contribute to decreasing this probability.

The solution of Eq. S11 with initial condition $g_{i}=0$at $u=t_{E}$ is

$g_{i}(u)=\frac{1-F_{i, 2}}{1-\frac{\tau_{i}}{T}}\left(e^{-\left[\frac{t_{E}-(t-x)}{\tau_{i}}+\frac{t-t_{E}}{T}\right]}-e^{- \frac{x}{\tau_{i}}}\right)$ [S12]

and the solution of Eq. S9 and Eq. S10 is

$f_{i}(u)=\left\{\begin{matrix} \frac{1-F_{i, 1}}{1-\frac{\tau_{i}}{T}}\left(e^{- \frac{u-(t-x)}{T}}-e^{- \frac{u-(t-x)}{\tau_{i}}}\right)\text{ if }t-x<u<t_{E} \\ \frac{1-F_{i, 1}}{1-\frac{\tau_{i}}{T}}\left(e^{- \frac{t_{E}-(t-x)}{T}}-e^{- \frac{t_{E}-(t-x)}{\tau_{i}}}\right)e^{-\frac{u-t_{E}}{T^{*}}}\text{ if }t_{E}<u<t \end{matrix}\right.$ [S13]

Clearly so that finally

$\begin{matrix} \forall i\text{ }1\leq i\leq N_{1} \\ R_{i}(x,t,t_{E})=e^{- \frac{x}{\tau_{i}}}+\frac{1-F_{i, 1}}{1-\frac{\tau_{i}}{T}}\left(e^{- \frac{t_{E}-(t-x)}{T}}-e^{- \frac{t_{E}-(t-x)}{\tau_{i}}}\right)e^{-\frac{t-t_{E}}{T^{*}}}+\frac{1-F_{i, 2}}{1-\frac{\tau_{i}}{T}}\left(e^{-\left[\frac{t_{E}-(t-x)}{\tau_{i}}+\frac{t-t_{E}}{T}\right]}-e^{- \frac{x}{\tau_{i}}}\right) \end{matrix}$ [S14]

The case of a module that is disease-permissive when naïve can be deduced (as in the simple case with constant environment, eqs. S1 to S8) from the previous case (protective when naïve) just by changing into and into so that:

$\begin{matrix} \forall i\text{ }N_{1}+1\leq i\leq N \\ 1-R_{i}(x,t,t_{E})=e^{- \frac{x}{\tau_{i}}}+\frac{F_{i, 1}}{1-\frac{\tau_{i}}{T}}\left(e^{- \frac{t_{E}-(t-x)}{T}}-e^{- \frac{t_{E}-(t-x)}{\tau_{i}}}\right)e^{-\frac{t-t_{E}}{T^{*}}}+\frac{F_{i, 2}}{1-\frac{\tau_{i}}{T}}\left(e^{-\left[\frac{t_{E}-(t-x)}{\tau_{i}}+\frac{t-t_{E}}{T}\right]}-e^{- \frac{x}{\tau_{i}}}\right) \end{matrix}$ [S15]

Now, according to Eq. S7

$1-R(x,t,t_{E})=\prod_{i=1}^{N_{1}} \left[1-R_{i}(x,t,t_{E})\right]\prod_{j=N_{1}+1}^{N} \left[1-R_{j}(x,t,t_{E})\right]$ [S16]

so that we finally get:

[S17]

As the affected modules cannot be specified at this time, we extend the mean-field approximation introduced above by assuming that the *N_1_* MDPs $F_{i,1}$ ($1\leq i\leq N_{1}$) are all identical and equal to their geometric mean $\Phi_{1}$, the *N-N_1_* MDPs $F_{j,1}$($N_{1}+1\leq j\leq N$) equal to $\Psi_{1}$, and the same for the new environment *E_2_* (replacing $\Phi_{1}$ by $\Phi_{2}$ and $\Psi_{1}$ by $\Psi_{2}$). The probability that CD occurs before age among people born at in a changing environment with a sharp transition from *E_1_* to *E_2_* at time is then written

[S18]
