# Supplementary material for: Network Modeling of Crohn’s Disease Incidence
Source: PLoS One. 2016 Jun 16;11(6):e0156138. doi: 10.1371/journal.pone.0156138 (PMC4911211; doi:10.1371/journal.pone.0156138)
Supplement: S4 File — (DOCX) [file pone.0156138.s004.docx]

**S4 File. Computation of the age-dependent incidence rate effect in a progressively varying environment.**

We now consider the progressive environmental change depicted in Figure A. For those individuals that are born at time *t-x* (cohort of age *x* at time *t*):

A fraction 1-ξ(t) remains under the old environment *E_1_* (denoted group I),

A fraction ξ(t-x) lives already under the new environment *E_2_* (denoted group II),

A fraction ξ(t) – ξ(t-x) transits from *E_1_* to *E_2_* (denoted group III).

Note: the cohort born at time *t-x* (i.e. during the year *t-x*) is assumed to be representative of the whole population, hence the fraction of individuals that are exposed to the new environment is the same among this cohort as in the whole population (also assuming that the environmental change affects individuals notwithstanding their age).

For time $t<t_{1}$ the environment (denoted $E_{1}$) was supposed constant and uniform (i.e. it did not change with time and it was homogenously distributed in the population). After the transition was completed, at time $t>t_{2}$, the new environment (denoted $E_{2}$) was constant and uniform again. For any time t between $t_{1}$ and $t_{2}$, we note $\xi\left(t\right)$ the fraction (increasing with time $t$) of the population that was exposed to the new environment $E_{2}$ ( $1-\xi\left(t\right)$ being the fraction of the population still exposed to environment $E_{1}$ at time t). Finally, we noted t_50_ the date at which half of the population was exposed to the environmental factor (Figure A).

We assumed that the environmental change did not significantly affect the overall stabilization dynamics and the biological network model, so that the averaged values $\tau$ and *N* remained unchanged with time. In order to model the impact of the environmental change on both parameters Φ and T we also assumed:

(i) the MDP $F_{i}$ of each module $M_{i}$ changes from $F_{i,1}$ to $F_{i,2}$ as the environment changes from $E_{1}$ to $E_{2}$ so that the geometrical mean Φ of the *N* modules changes from Φ_1_ to Φ_2_.

(ii) the value of the aging lifetime *transiently* changes form $T$ to $T^{*}$ as the environment changes from $E_{1}$ to $E_{2}$ *only* for those modules that are yet stabilized in the original environment $E_{1}$. For the others, still immature modules, the aging lifetime is set to the original value $T$. The rationale behind hypothesis (ii) is that the network may adapt to the new environment so far as concerns the immature modules. When these immature modules get stabilized in the new environment $E_{2}$ they are as robust to ageing as the former modules were in the original environment $E_{1}$.

The fraction of the cohort born at time $t-x$ that is ill at time t is written as:

$\Pr(CD\left|x,t\right.)=\left[1-\xi(t)\right]P_{I}(CD\left|x,t\right.)+\xi(t-x)P_{II}(CD\left|x,t\right.)+\int_{t-x}^{t} \frac{d\xi}{dt_{E}}\Pr(CD\left|x,t\right.,t_{E}) dt_{E}$ [S19]

with

$P_{I}(CD|x)=\Pr(CD\left|x,t,t)=\right.\left\{\left(1-\frac{1-\Phi_{1}}{1-\frac{\tau}{T}}\right)\left(1-e^{-\frac{x}{\tau}}\right)+\frac{1-\Phi_{1}}{1-\frac{\tau}{T}}\left(1-e^{-\frac{x}{T}}\right)\right\}^{N_{1}}\left\{\left(1-\frac{\Psi_{1}}{1-\frac{\tau}{T}}\right)e^{-\frac{x}{\tau}}+\frac{\Psi_{1}}{1-\frac{\tau}{T}}e^{-\frac{x}{T}}\right\}^{N-N_{1}}$[S20] $\mathrm{P}_{II}(CD|x)=\Pr(CD\left|x,t,t-x)=\right.\left\{\left(1-\frac{1-\Phi_{2}}{1-\frac{\tau}{T}}\right)\left(1-e^{-\frac{x}{\tau}}\right)+\frac{1-\Phi_{2}}{1-\frac{\tau}{T}}\left(1-e^{-\frac{x}{T}}\right)\right\}^{N_{1}}\left\{\left(1-\frac{\Psi_{2}}{1-\frac{\tau}{T}}\right)e^{-\frac{x}{\tau}}+\frac{\Psi_{2}}{1-\frac{\tau}{T}}e^{-\frac{x}{T}}\right\}^{N-N_{1}}$[S21]

and $\Pr\left(CD\left|x,t,t_{E}\right.\right)$ is given in Equation S18.

Note that $P_{I}(CD|x)=\Pr(CD\left|x,t,t)\right.$because $t_{E}=t$ for people of group I, whereas $P_{II}(CD|x)=\Pr(CD\left|x,t,t-x)\right.$ because $t_{E}=t-x$ for people of group II.

The age-specific incidence rate is:

$I(x,t)=\frac{\Pr(CD\left|x+\Delta t,t+\Delta t)-\Pr(CD\left|x,t)\right.\right.}{\Delta t}=\frac{\partial}{\partial x}\Pr(CD\left|x,t)\right.+\frac{\partial}{\partial t}\Pr(CD\left|x,t)\right.$ [S22]

so that we finally get

$I(x,t)=\left[1-\xi(t)\right]\frac{dP_{I}}{dx}(x)+\xi(t-x)\frac{dP_{II}}{dx}(x)+\int_{t-x}^{t} \frac{d\xi}{dt_{E}}\left[\frac{\partial}{\partial x}\Pr(CD\left|x,t,t_{E})+\frac{\partial}{\partial t}\Pr(CD\left|x,t,t_{E})\right.\right.\right]$ [S23]


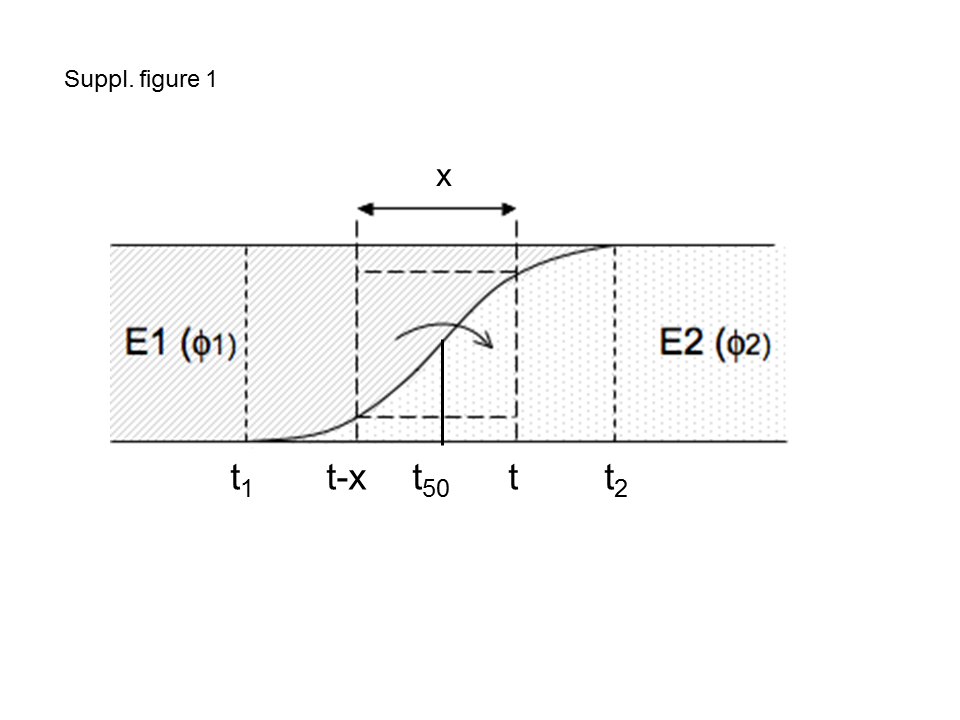


**Figure A.** Schematic representation of the transition between two environments E_1_ and E_2_ with a progressive exposure of the general population. t_1_ and t_2_ are the dates of the beginning and the end of the transition, respectively. Before t_1_ and after t_2_, the environmental exposure is supposed to be stable (constant with time) and uniformly distributed in the population. During the transition, an increasing proportion of the population was exposed to the environmental risk factor(s). t_50_ represents the date when half of the population has been exposed to the risk factor(s). During the transition, at any time t of exposure, the cohort born at time t-x can be divided into 3 subgroups:

i) A fraction of people who only lived in environment E_1_

ii) A fraction of people who only lived in environment E_2_

iii) A fraction of people for whom environment has changed

Environments E_1_ and E_2_ were associated with module disease propensities Φ_1_ (also referred as Φ_before_) and Φ_2_ (also referred as Φ_after_).
