# Supplementary material for: Network Modeling of Crohn’s Disease Incidence
Source: PLoS One. 2016 Jun 16;11(6):e0156138. doi: 10.1371/journal.pone.0156138 (PMC4911211; doi:10.1371/journal.pone.0156138)
Supplement: S5 File — (DOCX) [file pone.0156138.s005.docx]

**S5 File. Computation of the distribution of the OR.**

We first condition on the set of loci$\alpha$ which have a prescribed RAF equal to *p*. Then, we observe that

$RR(\alpha)=H_{p,\Phi}\left[F(\alpha_{R})\right]$ [S32]

with $H_{p,\Phi}(x)=\frac{x(1-p)^{2}}{\Phi-x(2p-p^{2})}$ [S33]

Therefore, if $RR(\alpha)$is distributed according to some probability density $\nu_{p}$ then, applying the change of variable formula, one deduces that $F(\alpha_{R})$ is distributed according to the density:

$g_{p}(x)=\nu_{p}\left(\frac{x(1-p)^{2}}{\Phi-x(2p-p^{2})}\right)\frac{\Phi(1-p)^{2}}{\left[\Phi-x(2p-p^{2})\right]^{2}}$ [S34]

We remark from the data that $\nu_{p}$ actually depends very weakly upon *p*(suppl. figure 2). We thus assume that $\nu_{p}=\nu$ is independent of *p*. Denoting *ρ(p)* the RAF probability distribution, we reconstruct the final density for $F(\alpha_{R})$ across all loci by:

$g(x)=\int\nu\left(\frac{x(1-p)^{2}}{\Phi-x(2p-p^{2})}\right)\frac{\Phi(1-p)^{2}}{\left[\Phi-x(2p-p^{2})\right]^{2}}\rho(p)dp$ [S35]

**Suppl. figure 2.** Odds Ratios and risk allele frequencies in the general population for the disease-associated alleles reported for CD (derived from Jostins L et al. 2012).
