# Supplementary material for: Network Modeling of Crohn’s Disease Incidence
Source: PLoS One. 2016 Jun 16;11(6):e0156138. doi: 10.1371/journal.pone.0156138 (PMC4911211; doi:10.1371/journal.pone.0156138)
Supplement: S6 File — (DOCX) [file pone.0156138.s006.docx]

**S6 File. Extrapolation of the OR distribution from an available data set (ref 2).**

Due to power limitations inherent to GWAS, the published distributions of ORs are truncated for the lowest values. For each locus, the OR can be estimated by the value y=ad/bc from the following contingency table where the at-risk genotypes correspond to (α_R_, α_R_) and (α_R_, α_P_) and the protective genotype corresponds to (α_P_, α_P_)):

|  | cases | controls |
| --- | --- | --- |
| At risk | a | b |
| Protected | c | d |

(NB: In ref 2, the estimates were based on a sample of $N_{1}=20 700$ CD cases and N_2_ = 37 747 healthy controls).

Following Woolf’s method, y is a random variable that can be regarded as distributed according to a log-normal law : ln(y) ~ *N*(ln(x), σ^2^) where the mean, i.e. ln(x), is the natural logarithm of the “true” OR in the general population and the standard deviation σ is given by:

$$\sigma=\sqrt{\frac{1}{a}+\frac{1}{b}+\frac{1}{c}+\frac{1}{d}}$$

Thus :

$P(y)dy=\frac{1}{\sigma\sqrt{2\pi}}e^{-\frac{\left(\ln y-\ln x\right)^{2}}{2\sigma^{2}}}d(\ln y)$ [S36]

An estimate is considered as statistically significant if *y* >1+ε where ε corresponds to the specified threshold of significance (P<5.10^-8^ in ref 2 corresponding to ε = 5.325 σ). Thus the probability to measure a significant estimate of the OR for a “true” value x in the general population is:

$P(x)=\int_{\ln(1+\varepsilon)}^{\infty} \frac{1}{\sigma\sqrt{2\pi}}e^{-\frac{\left(\ln y-\ln x\right)^{2}}{2\sigma^{2}}}d(\ln y)=\int_{\varepsilon}^{\infty} \frac{1}{\sigma\sqrt{2\pi}}e^{-\frac{\left(u-\ln x\right)^{2}}{2\sigma^{2}}}du$ [S37]

Let ν(x) be the probability distribution of the “true” OR in the general population over all genetic loci. Then

$\int_{1}^{\infty} \nu(x)dx=1$ [S38]

The histogram of these “true” OR can be fitted by a continuous interpolating function H(x)=N_SNP_ν(x)Δ with N_SNP_ being the total number of studied loci (10^5^ < N_SNP_ < 10^6^ loci in ref 2) and Δ the histogram bin width. The total number of “true” OR values in the bin [x-Δ/2, x+Δ/2] is equal to H(x). Now the probability that a given OR in this bin is measured as significant is equal to P(x). Hence the mean number of OR that are measured as significant in this bin is equal to

$N(x)=P(x)H(x)$ [S39]

The number of significant OR that has been actually measured (derived from Jostins 2012) in the bin [x-Δ/2, x+Δ/2] is N_mes_(x). This histogram is shown in figure 6a. The value N_mes_(x) is an estimation of N(x). Therefore the corrected histogram H(x) is estimated by the ratio

$H(x)=\frac{N_{mes}(x)}{P(x)}$ [S40]

This extrapolated histogram is drawn in figure 6a.
